# Propagation of a hospital-associated cluster of COVID-19 in Malaysia

**DOI:** 10.1186/s12879-021-06894-y

**Published:** 2021-12-09

**Authors:** Diane Woei-Quan Chong, Vivek Jason Jayaraj, Chiu-Wan Ng, I-Ching Sam, Mas Ayu Said, Rafdzah Ahmad Zaki, Noran Naqiah Hairi, Nik Daliana Nik Farid, Victor Chee-Wai Hoe, Marzuki Isahak, Sasheela Ponnampalavanar, Sharifah Faridah Syed Omar, Shahrul Bahyah Kamaruzzaman, Hang-Cheng Ong, Kejal Hasmukharay, Nazirah Hasnan, Adeeba Kamarulzaman, Yoke Fun Chan, Yoong Min Chong, Sanjay Rampal

**Affiliations:** 1 Centre for Epidemiology and Evidence-based Practice, Department of Social and Preventive Medicine, Faculty of Medicine, University of Malaya, 50603 Kuala Lumpur, Malaysia; 2 Ministry of Health Malaysia, 62590 Putrajaya, Malaysia; 3 Department of Medical Microbiology, Faculty of Medicine, University of Malaya, 50603 Kuala Lumpur, Malaysia; 4 Department of Medicine, Faculty of Medicine, University of Malaya, 50603 Kuala Lumpur, Malaysia; 5 Department of Rehabilitation Medicine, Faculty of Medicine, University of Malaya, 50603 Kuala Lumpur, Malaysia

**Keywords:** SARS-CoV-2, Epidemiological investigation, Whole genome sequencing, Hospital, Infection control, Multidisciplinary

## Abstract

**Background:**

Hospitals are vulnerable to COVID-19 outbreaks. Intrahospital transmission of the disease is a threat to the healthcare systems as it increases morbidity and mortality among patients. It is imperative to deepen our understanding of transmission events in hospital-associated cases of COVID-19 for timely implementation of infection prevention and control measures in the hospital in avoiding future outbreaks. We examined the use of epidemiological case investigation combined with whole genome sequencing of cases to investigate and manage a hospital-associated cluster of COVID-19 cases.

**Methods:**

An epidemiological investigation was conducted in a University Hospital in Malaysia from 23 March to 22 April 2020. Contact tracing, risk assessment, testing, symptom surveillance, and outbreak management were conducted following the diagnosis of a healthcare worker with SARS-CoV-2 by real-time PCR. These findings were complemented by whole genome sequencing analysis of a subset of positive cases.

**Results:**

The index case was symptomatic but did not fulfill the initial epidemiological criteria for routine screening. Contact tracing suggested epidemiological linkages of 38 cases with COVID-19. Phylogenetic analysis excluded four of these cases. This cluster included 34 cases comprising ten healthcare worker-cases, nine patient-cases, and 15 community-cases. The epidemic curve demonstrated initial intrahospital transmission that propagated into the community. The estimated median incubation period was 4.7 days (95% CI: 3.5–6.4), and the serial interval was 5.3 days (95% CI: 4.3–6.5).

**Conclusion:**

The study demonstrated the contribution of integrating epidemiological investigation and whole genome sequencing in understanding disease transmission in the hospital setting. Contact tracing, risk assessment, testing, and symptom surveillance remain imperative in resource-limited settings to identify and isolate cases, thereby controlling COVID-19 outbreaks. The use of whole genome sequencing complements field investigation findings in clarifying transmission networks. The safety of a hospital population during this COVID-19 pandemic may be secured with a multidisciplinary approach, good infection control measures, effective preparedness and response plan, and individual-level compliance among the hospital population.

**Supplementary Information:**

The online version contains supplementary material available at 10.1186/s12879-021-06894-y.

## Background

Severe acute respiratory syndrome coronavirus 2 (SARS-CoV-2) is a highly transmissible virus. Hospitals became potential disease transmission hubs due to a surge in admissions of COVID-19 patients [1]. During the early phase of the pandemic, the burden of intrahospital transmission was evident within a study in China that reported a 41% prevalence of hospital-associated COVID-19 infections among patients [2].

As a result, much focus was placed on preventing hospitals from becoming potential loci of outbreaks by protecting healthcare workers (HCWs) from the patient-to-HCW transmission of disease and the use of personal protective equipment (PPE) [3–5]. However, hospital-associated cases of COVID-19 often demonstrate complex transmission networks involving HCWs, patients, and the community [6, 7]. The HCWs may acquire the infection not just from COVID-19 patients but also from other HCWs. Additionally, HCWs are the interface between the hospital setting and community [6]. Due to widespread community transmission, they are more likely to acquire COVID-19 outside the hospital setting [3, 8].

Intrahospital transmission of the disease threatens the healthcare systems as it increases morbidity and mortality among the patients. Thus, it is imperative to deepen our understanding of transmission events in hospital-associated cases of COVID-19 [6] for timely implementation of infection prevention and control measures in the hospital and avoid future outbreaks. However, limited evidence is available in understanding the transmission of SARS-CoV-2 in the hospital setting [6]. Epidemiological outbreak investigation provides spatial and temporal information and possible transmission routes, while whole genome sequencing (WGS) provides complementary data supporting or refuting epidemiologically linked transmission among the cases. Integrating epidemiological outbreak investigation with WGS, in particular, could increase confidence in identifying whether the infection is hospital or community-acquired [7]. This study examined the use of epidemiological case investigation combined with WGS of cases to investigate and manage a hospital-associated cluster of COVID-19 cases.

## Methods

### Study setting and participants

University Malaya Medical Centre (UMMC) in Kuala Lumpur, Malaysia, serves an approximate 1.12 million patients annually. It was one of Malaysia’s 35 designated COVID-19 hospitals [9]. A multidisciplinary task force was established in the hospital in responding to the pandemic. The task force's responsibilities include: (1) resource management; (2) reviewing national policies on the prevention, treatment, and control of COVID-19; (3) developing hospital-specific guidelines; (4) implementing infection prevention and control measures; and (5) providing training and psychological support to HCWs. A sub-committee of the task force, consisting of specialists in the field of public health, occupational safety and health, infectious disease, infection control, and microbiology, were tasked with preventing COVID-19 transmission within the hospital. The COVID-19 preparedness and response plan included five key components: case notification, contact tracing, risk assessment and testing, HCWs and patients surveillance, and outbreak management to ensure the safety of the HCWs and patients [9].

The hospital received notification of a HCW tested positive for SARS-CoV-2 on 23 March 2020. The index case (referred to as AH1 henceforth) was in contact with patients and HCWs two days before and six days after the onset of symptoms. Following notification of the positive case, contact tracing of HCWs and patients exposed to AH1 in a non-COVID-19 ward and various hospital events attended by AH1 was initiated. The outbreak involved HCWs and patients from a non-COVID-19 ward and the emergency department. None of the cases had undergone previous SARS-CoV-2 testing. The taskforce monitored the cluster from the detection of the first case up until two weeks after the last diagnosed case. The outbreak investigation was conducted from 23 March to 22 April 2020. The cumulative incidence of COVID-19 in Malaysia was 4.1 and 17.1 per 100,000 population between 23 March and 22 April 2020. Meanwhile, the COVID-19-associated death rate was 0.03 and 0.29 per 100,000 population during the same time [10]. 

### Case definitions

In this study, a confirmed case of COVID-19 was defined as an individual with laboratory confirmation of infection through the detection of SARS-CoV-2 nucleic acid in upper respiratory specimens from nasopharyngeal and oropharyngeal swabs.

A hospital-associated cluster was defined as two or more confirmed COVID-19 cases among HCWs, patients, and the community within 14 days. These cases had exposures reported at a common event or locations within the hospital (hospital-associated) or in the community (community-associated). In this study, any COVID-19 case reported among the HCWs (HCW-case) that could be spatially and temporally linked to AH1 and the subsequent cases were included. The patients were included based on hospital-onset or community-onset of disease (patient-case). Patients with hospital-onset of COVID-19 included patients who were diagnosed with COVID-19 after 48 hours of admission to the hospital. Meanwhile, patients with community-onset COVID-19 were diagnosed with COVID-19 after 48 hours of hospital admission and had earlier healthcare contact within the last 14 days [7]. Additionally, community-associated cases (community-case) that could be linked to an HCW-case or patient-case without any history of healthcare contact within the last 14 days were included to demonstrate the dynamic interactions between HCWs and patients within the hospital setting and the community. Within the hospital-associated cluster, the index case was defined as the first case that was tested positive for COVID-19. First and second generation cases were those who were tested positive after contact with either the index case or a first generation case, respectively. Symptomatic cases included those who developed COVID-19 signs and symptoms such as fever, cough, sore throat, anosmia, gastrointestinal symptoms and lethargy during the entire admission period. Meanwhile, asymptomatic cases were defined as those who did not report any symptoms during their infections [11].

### Epidemiological investigation

Contact tracing was initiated within 24 h of notification of any HCW-case or patient-case testing positive for SARS-CoV-2. Close contact was defined as any individual within one meter of a COVID-19 case for at least 15 min from two days before and up to 14 days after symptom onset of the case [9]. It included HCWs providing direct care for COVID-19 patients or working with other HCWs infected with COVID-19 [12]. Additionally, patients who had direct physical contact with a confirmed case or shared a cubicle, treatment or procedure area, or bathroom with a confirmed case were included as close contacts, as were patients who had unprotected direct contact with infectious secretions.

The possible infectious period was defined as two days before the onset of symptoms and 14 days following [13]. A two-stage interview procedure was used to trace the contacts. Forward contact tracing was conducted 48 hours before symptom onset until isolation to identify newly exposed HCWs and patients. Backward contact tracing was carried out 14 days before symptom onset to identify the potential source of infection. Furthermore, information such as cases’ demographic characteristics, date of onset of symptoms, and their clinical activities during this period was gathered.

The hospital’s electronic medical records (EMR) also aided in contact tracing. EMR was used to supplement the information obtained from interviewing the HCWs, trace patients’ movements throughout their care process, and identify HCWs who interacted with them during the infectious period. Meanwhile, outside the hospital setting, close contacts of HCWs and patients were identified and notified to the district health offices for screening, home quarantine, and surveillance.

All HCWs exposed to a COVID-19 case had their risk of exposure assessed and stratified into baseline or no identifiable, low-, medium-, or high-risk [see Additional file 1]. Risk assessment was conducted using a standardized questionnaire that included questions about the following: (1) the duration and type of exposure; (2) the case’s clinical symptoms and whether the case wore a face mask; (3) the presence of an aerosol-generating procedure; and (4) the type of PPE worn by the HCW. In addition, they had SARS-CoV-2 samples taken and were quarantined based on their risk levels. All HCWs with higher than baseline risk were placed under active daily symptoms surveillance for 14 days from the last day of exposure to a case. HCWs who developed new or worsening symptoms were reassessed and, if warranted, tested for SARS-CoV-2. The management of HCWs following the exposure to a positive case based on risk level is described elsewhere [14].

Patients with COVID-19 exposure were quarantined. If they developed symptoms, they were screened for SARS-CoV-2. These patients were allowed to complete the 14-day quarantine at home if they were fit to be discharged and had a negative COVID-19 test before discharge. Meanwhile, patients discharged home before the confirmed case’s identification were requested to return for screening, and district health offices were notified for home quarantine and symptom surveillance. All confirmed COVID-19 cases were isolated and treated in the hospital [15]. Based on the initial recommendation by the World Health Organization (WHO), these patients were discharged from the hospital after they had recovered clinically and had two negative real-time polymerase chain reaction (PCR) results on sequential samples taken at least 24 hours apart [16]. 

### Laboratory and bioinformatics methods

Nasopharyngeal and oropharyngeal swabs were obtained from patients with suspected COVID-19. Real-time PCR detection of SARS-CoV-2 was performed with a WHO-recommended protocol [17], Allplex 2019-nCoV Assay (Seegene, Korea), or abTES COVID-19 qPCR I Kit (AITbiotech, Singapore). WGS was directly carried out with the iSeq 100 system (Illumina, USA) for 21 selected patients using their earliest available samples with the highest viral load, with the ARTIC-nCoV-2019 protocol [18]. The samples were selected based on criteria including availability of stored sample, PCR cycle threshold value less than 32, cases from different age groups, unclear epidemiological link between the cases, or if a single representative for a clear subcluster (e.g., a household) was deemed sufficient. The earliest available sample during the illness with the highest viral load was selected for genome sequencing for each selected case.

Methodology and the resulting sequences have been previously reported [19]. The sequenced reads were edited and mapped to reference strain Wuhan-Hu-1 (GenBank accession number MN908947) using Geneious Prime 2020 (Biomatters, New Zealand). Genetic lineages of the sequences were determined with the Pangolin COVID-19 Lineage Assigner (www.pangolin.cog-uk.io) [20]. The sequences were aligned with 54 other Malaysian genome sequences of the same lineage available at the Global Initiative on Sharing All Influenza Data website (GISAID; www.gisaid.org) as of 20 September 2020. Phylogenetic analysis was carried out with RAxML 8.2.11 in Geneious with default parameters (generalized time-reversal + gamma substitution model and 1000 bootstrap replications).

### Statistical methods

Demographic and case characteristics were described using frequency (percentage) and median (interquartile range, IQR). An epidemic curve was constructed based on the dates of symptom onset and stratified by transmission setting. A timeline of infection-related events and the simplified networks along which transmission propagated were visualized using a dendrogram. The incubation period and serial interval were empirically estimated using a likelihood function by fitting a log-normal and gamma distribution. Markov-chain Monte Carlo resampling was used to estimate the 95% confidence interval [21, 22]. All statistical analyses were carried out using R version 3.6.0 [23]. A two-sided P < 0.05 was considered statistically significant.

## Results

Investigation of a hospital-associated cluster between 23 March and 22 April 2020 suggested an epidemiological link between 38 cases of COVID-19 admitted to UMMC. However, molecular investigation based on phylogenetic analysis revealed that four of the 38 cases were not related to the hospital-associated cluster. Therefore, these four cases were excluded from further analysis. Contact tracing identified 350 HCWs and 93 patients as close contacts of AH1 and subsequent cases. The proportion of HCWs and patients tested positive was 4.1%. Among the hospital-associated contacts, 2.6% and 9.7% of HCWs and patients were tested positive for SARS-CoV-2, respectively. Risk assessment information was missing for 50 of the HCWs (14.3%) as manual reporting mechanisms were implemented in the hospital during the outbreak. The risk levels of the remaining 300 HCWs were categorized as low-risk (57.0%), medium-risk (23.0%), and high-risk (20.0%) exposures.

This cluster included 34 cases that involved ten HCW-cases, nine patient-cases, and 15 community-cases (Table 1). Five of the nine patient-cases had community-onset of COVID-19 (Additional file 2). The median (IQR) age of the cases was 42.0 years (29.0 to 80.8 years). The majority of the cases involved females (73.5%). Fever (50.0%), cough (47.1%), and sore throat (29.4%) were the most frequently reported symptoms. More than half of the cases had previous comorbidities (55.9%). All five deaths (14.7%) occurred in adults above the age of 60. The median duration between symptom onset and diagnosis was 2.0 (1.0 to 5.0) days. The median duration between symptom onset and the diagnosis was 3.0 (2.0 to 6.0) days among the HCW-cases, which was higher than that in patient-cases [1.0 (0.0 to 2.0)] and the community-cases [1.0 (0.0 to 3.5)]. However, there were no statistically significant differences between the groups (p = 0.10). The cases were admitted to the hospital within a median of 2.0 (1.0 to 4.0) days of diagnosis. The median duration of hospital stay was 20.0 (14.0 to 32.0) days. The outbreak investigation identified six asymptomatic cases (17.6%) (Table 1).

**Table 1:** Demographic and epidemiological characteristics of COVID-19 cases

	Overall	Cases		
		Healthcare workers	Patients	Community
Count, n	34	10	9	15
Age* in years, median [IQR]	42.0 [29.0, 80.8]	31.0 [28.0, 37.0]	80.0 [74.0, 81.0]	36.0 [28.5, 85.0]
Age* more than 60 years (%)	15 (44.1)	0 (0.0)	9 (100.0)	6 (40.0)
Female (%)	25 (73.5)	8 (80.0)	6 (66.7)	11 (73.3)
Symptomatic (%)	28 (82.4)	10 (100.0)	7 (77.8)	11 (73.3)
Fever	17 (50.0)	6 (60.0)	5 (55.6)	6 (40.0)
Cough	16 (47.1)	5 (50.0)	4 (44.4)	7 (46.7)
Sore throat*	10 (29.4)	7 (70.0)	1 (11.1)	2 (13.3)
Coryza	4 (11.8)	3 (30.0)	–	1 (6.7)
Anosmia	3 (8.8)	3 (30.0)	–	–
Dyspnoea	3 (8.8)	1 (10.0)	1 (11.1)	1 (6.7)
Gastrointestinal symptoms	4 (11.8)	–	3 (33.3)	1 (6.7)
Lethargy	1 (2.9)	–	–	1 (6.7)
Previous comorbidities* (%)	19 (55.9)	3 (30.0)	9 (100.0)	7 (46.7)
Diabetes mellitus	12 (35.3)	–	7 (77.8)	5 (33.3)
Hypertension	10 (29.4)	–	5 (55.6)	5 (33.3)
Cardiovascular disease	6 (17.6)	–	5 (55.6)	1 (6.7)
Hyperlipidaemia	6 (17.6)	–	6 (66.7)	–
Chronic kidney disease	4 (11.8)	–	3 (33.3)	1 (6.7)
History of cancer	2 (5.9)	1 (10.0)	–	1 (6.7)
Other comorbidities	14 (41.2)	2 (20.0)	7 (77.8)	5 (33.3)
Duration (in days), median [IQR]				
Symptom onset to diagnosis	2.0 [1.0, 5.0]	3.0 [2.0, 6.0]	1.0 [0.0, 2.0]	1.0 [0.0, 3.5]
Symptom onset to admission to hospital	2.0 [1.0, 4.0]	4.0 [2.0, 7.0]	1.0 [0.0, 2.0]	2.0 [0.0, 4.0]
Diagnosis to admission to hospital	0.0 [0.0, 1.0]	1.0 [0.0, 1.0]	0.0 [0.0, 0.0]	0.0 [0.0, 1.0]
Admission to discharge (n = 29)	20.0 [14.0, 32.0]	16.0 [9.0, 25.0]	33.0 [33.0, 39.0]	18.0 [11.0, 24.0]

Results are presented in median [interquartile range] for continuous variables and in the counts (percentages) for categorical variables

Gastrointestinal symptoms included nausea, vomiting, diarrhoea, and abdominal pain

Hospital-associated cases included HCW-cases and patient-cases. Among the patient-cases, there were five patients with community-onset of COVID-19 and four patients with hospital-onset of COVID-19. Community-cases were those that were community-associated (i.e., household and nursing home)

*A statistically significant difference was detected for age (p < 0.05), sore throat (p = 0.02), and previous comorbidities (p < 0.05)

**Fig. 1:**
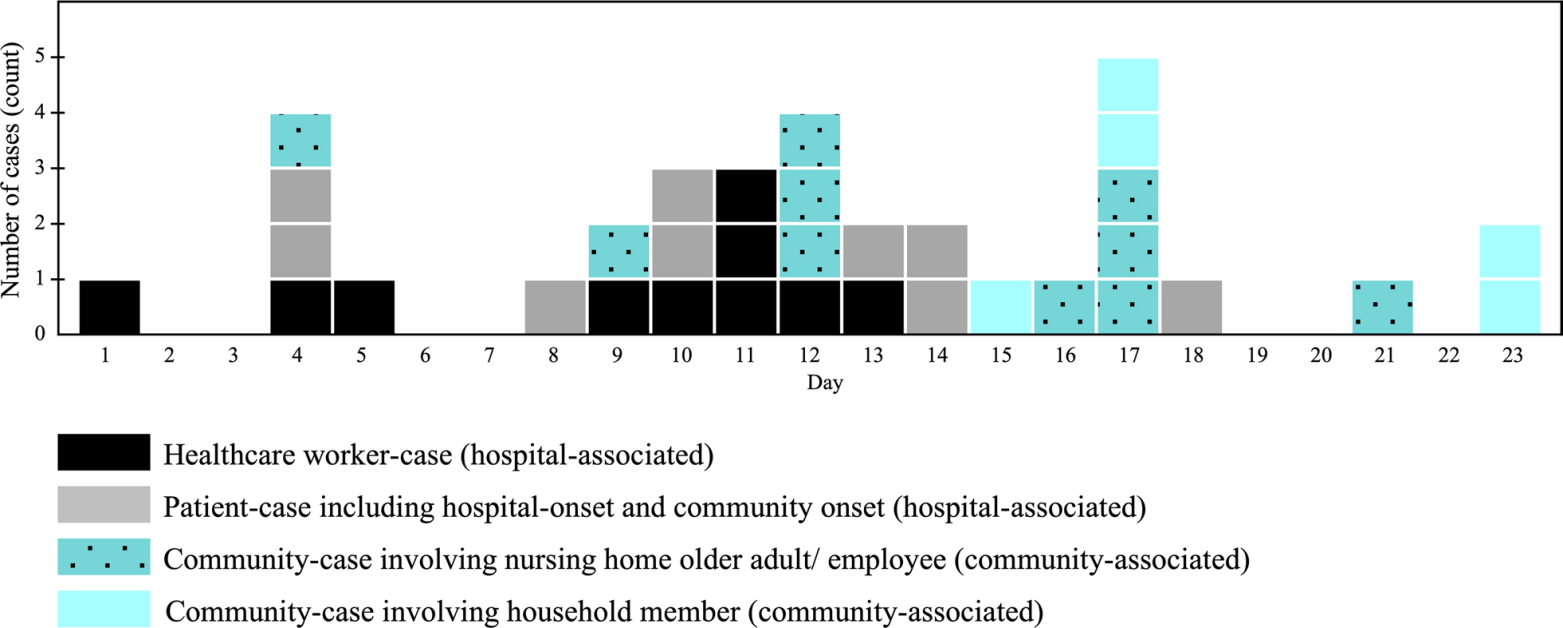
Epidemic curve of 34 COVID-19 cases based on symptom onset. Day 1 represents the day of symptom onset for the index case (17 March 2020). The dates of diagnosis based on real time-PCR are presented for the six cases who remained asymptomatic throughout the infection. Cases are stratified by location and category of case

The epidemic curve demonstrated a propagated outbreak with three peaks (Fig. 1). The outbreak started on 17 March 2020 (day 1). The first wave saw the majority of cases being hospital-associated, with a peak on day 4. From day 9, hospital- and community-associated cases occurred concurrently. After day 11, the decrease in hospital-associated cases was followed by a rise in community-associated cases, which peaked on day 12. From day 12 on, the outbreak primarily comprised community-associated cases from two households and a nursing home for older adults, reaching a peak on day 17. The outbreak ended on day 23, with the final two cases occurring among household members.

**Fig. 2:**
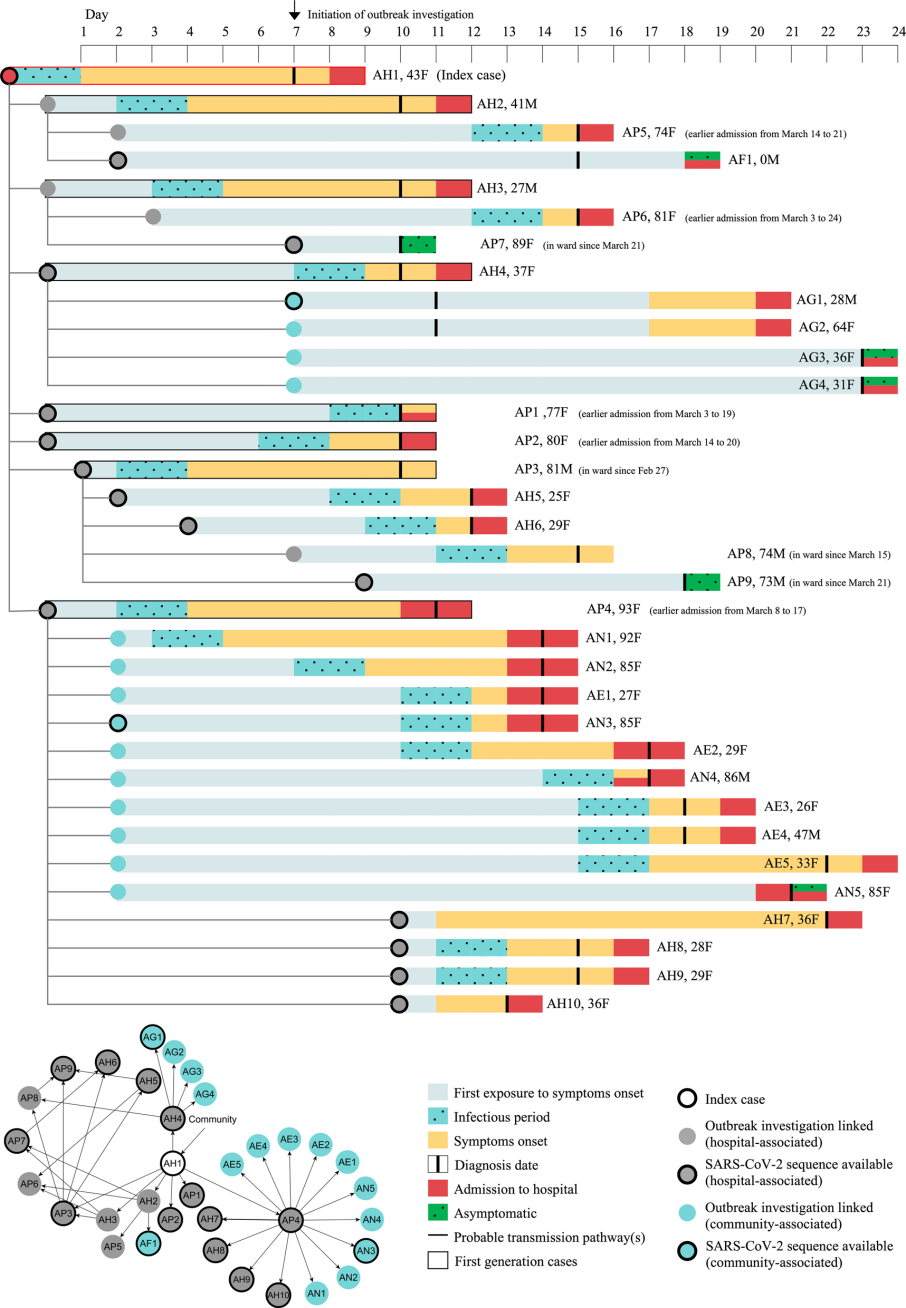
Timeline of events based on first exposure to a COVID-19 case, incubation period, diagnosis, and admission to the hospital from 15 March to 9 April 2020. Data of 34 cases are presented here. Top panel: Each row represents a case, and the rows are connected by lines to demonstrate the likely transmission of disease between case and contact. Each individual (case) is labeled based on transmission location (AH healthcare worker; AP- hospital patient; AF and AG- household members; AN- nursing home older adults; AE- nursing home employees) and number, age (years), and sex (F = female; M = male). AF1 was a one-month-old infant. AP3, AP7, AP8, and AP9 were patient-cases who were tested positive for SARS-CoV-2. Deaths occurred among AP1, AP3, AP4, AP7, AN4. The bottom left panel demonstrates multiple encounters between the cases and contacts

Figure 2 depicts the timeline of events and transmission chains of the 34 COVID-19 cases in the hospital-associated cluster. AH1 developed sore throat and fever on 17 March 2020. However, AH1 continued to work despite symptoms consistent with influenza, as AH1 had no known exposure to a confirmed COVID-19 case at the same time and sought treatment following the onset of anosmia. Six days after symptom onset, AH1 was tested and found to be positive for SARS-CoV-2. By day 11, contact tracing and close contact testing for AH1 identified seven additional cases, including three HCW-cases (AH2 to AH4) and four patient-cases (AP1 to AP4). By day 18, another two HCW-cases (AH5 and AH6) and five patient-cases (AP5 to AP9) had been infected with SARS-CoV-2. They were the second generation cases as these seven cases had no reported contact with AH1. Disease transmission was also reported among their household members, affecting one household member of AH2 (AF1) and four AH4 household members (AG1 to AG4).

Meanwhile, a secondary cluster was associated with patient-case AP4, who was exposed to an asymptomatic AH1 on day 0. AP4 was discharged two days later and spent ten days in a nursing home before being readmitted to the hospital with shortness of breath. On day 11, AP4 was diagnosed with COVID-19. AP4 was linked to ten community-cases at the nursing homes (residents AN1-5 and employees AE1-5) and four HCW-cases (AH7-10) who had close contact with AP4 during readmission to the hospital (Fig. 2). The median incubation period and serial intervals were 4.7 (95% CI: 3.5–6.4) days and 5.3 (95% CI: 4.3–6.5) days.

**Fig. 3:**
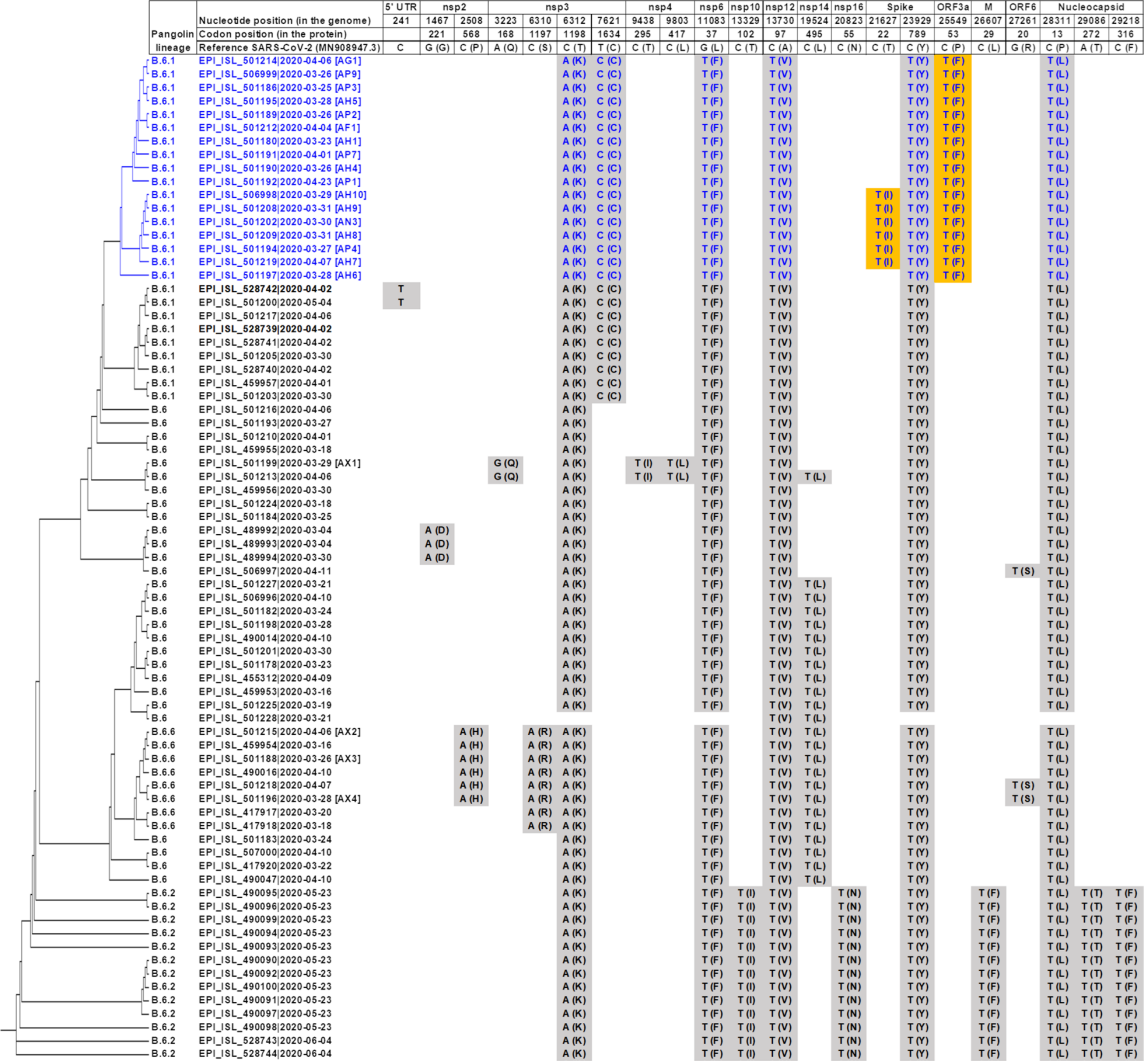
Phylogenetic tree and nucleotide and amino acid changes of 75 Malaysian SARS-CoV-2 sequences from the B.6 lineage. Sequences available in GISAID up to 20 September 2020 are included and compared to the reference strain Wuhan-Hu-1 (GenBank accession number MN908947). Sequences are named as “GISAID reference number|date of sample [case code].” Amino acids changes are shown in brackets. Sequences from 17 cases associated with the outbreak are shown in blue, with unique substitutions highlighted in orange. Sequences from 4 cases that were investigated but not considered part of this outbreak are indicated by their case numbers (AX1 to AX4). Only non-synonymous substitutions present in > 1 sequence are shown

In total, SARS-CoV-2 from 21 selected cases out of the 38 initially linked to the cluster underwent successful WGS (Additional file 3). All 21 sequences were from the B.6 lineage, which was the predominant lineage in Malaysia at the time, accounting for 75 (65.2%) of 115 publicly available sequences in GISAID but rarely (1.4%) amongst global sequences, as of 20 September 2020 [19]. When all Malaysian B.6 lineage sequences were analyzed, 17 of the study sequences (including the index case, AH1) clustered together with a distinctive non-synonymous mutation C25549T (P53F) in the ORF3a protein, which was not present in other Malaysian sequences (Fig. 3). These 17 sequences have 0–1 amino acid differences between each other and have been assigned to sublineage B.6.1. Six sequences (AP4, AN3, and AH7 to AH10) from the secondary cluster from case AP4 formed a separate phylogenetic group within the main cluster, with an additional, unique mutation C21627T (T22I) in the spike protein. Four study sequences (AX1 to AX4) had a further 6–7 amino acid differences and did not group with the primary cluster; further examination of the epidemiological data revealed contact with other cases outside of this cluster. These were the four excluded from this cluster.

HCWs, patients, and community contacts of the cases were traced, tested, and completed 14 days of symptoms surveillance. The infection control decision to restrict the affected ward to new admissions and strengthen surveillance of all patients who came into contact with the clinical areas prevented disease propagation. The following infection control and prevention measures were immediately implemented at the affected clinical areas: (1) decontamination and terminal cleaning of clinical and non-clinical areas occupied by the confirmed cases; (2) cohorting and limiting movements of HCWs and patients; (3) restricting visitors and caregivers to the hospital; (4) reinforcement of hand hygiene practices; (5) use of appropriate PPE based on risk assessment when providing patient care; and (6) disinfection of high touch areas. Other control and preventive measures included: (1) daily roll call for all HCWs to check for symptoms; (2) reminders to maintain physical distance, wear a mask at all times while in the hospital and avoid congregating with other HCWs during formal and informal interactions; and (3) risk communication to HCWs, patients and the public. After the last reported case on 9 April 2020, no additional cases were detected.

## Discussion 

This study integrated conventional epidemiological investigation and WGS to track COVID-19 transmission networks within a hospital-associated cluster that involved hospital and community transmission of COVID-19. We have demonstrated the complexity of SARS-CoV-2 transmission networks within a hospital-associated cluster involving HCWs, patients, and the community. Understanding disease transmission within the hospital setting supported the hospital in implementing outbreak management strategies during the ongoing outbreak, mainly to prevent further propagation of disease in the hospital and onward transmission of disease from infected HCWs and patients to the community. It allowed the hospital to improve its infection prevention and control measures in responding to multiple surges of COVID-19 cases that occurred in waves and in preventing future outbreaks in the hospital [6, 7].

In the hospital setting, the proportion of HCWs and patients tested positive was below 5.0%. The study demonstrated the importance of contact tracing, risk assessment and testing, symptom surveillance, and outbreak management in the hospital, particularly during an established outbreak in the healthcare setting. The cluster was brought under control through ongoing surveillance and containment measures activated early in the event of an HCW infected with COVID-19. Despite limited resources, the hospital remained vigilant and tested the HCWs and patients upon being identified as close contact. Repeated testing was carried out at day 13 following an exposure, upon symptom onset, or prior discharge to isolate the cases and break the chain of transmission promptly. Additionally, a broad definition of close contact was applied, and it enabled HCWs and patients with possible exposure to a COVID-19 case to be identified. They were tested, underwent symptom surveillance, and promptly removed from the workplace if their risk levels were identified as medium- or high-risk [14]. These approaches were valuable in preventing the further propagation of COVID-19 in the hospital setting. Meanwhile, district health offices were notified of community contacts of COVID-19 cases for SARS-CoV-2 testing. It is critical to highlight that the outbreak occurred during a period of statewide lockdown in Malaysia, which restricted individual movement within the community, likely contributing to the interruption of transmission within the community. Additionally, on 7 May 2020, the Malaysian Ministry of Health declared the cluster over [24].

There was no known transmission by the asymptomatic cases that were detected in this study. However, there is an increasing body of evidence that asymptomatic cases may contribute to the transmission of SARS-CoV-2 [25, 26]. Therefore, in this study, all close contacts were traced, quarantined, and placed under surveillance for 14 days from the last exposure, regardless of their symptomatic status. Although an initial period of six days between symptom onset and the index case diagnosis was observed in our investigation, we reduced the time gap to a median of two days for the remaining cases. The shorter time interval between the onset of symptoms and diagnosis of the cases was likely due to the effective implementation of outbreak management strategies in the hospital and the close collaboration with district health offices for contact tracing and surveillance of community contacts. Meanwhile, the median period between symptom onset and diagnosis appeared longer for HCWs (P > 0.05). Reasons for this included: (1) chance; (2) HCWs’ lack of healthcare-seeking during this first initial large outbreak in the hospital; and (3) contact tracing, risk assessment, and test scheduling introduced an administrative delay. Following this outbreak, the hospital emphasized daily self-reporting of symptoms and encouraged HCWs to be vigilant in seeking healthcare.

The hospital-associated cluster demonstrated the difficulty of managing an outbreak such as COVID-19 in a hospital setting when sustained community transmission had occurred. Between March and April 2020, Malaysia experienced its second wave of infections, and local transmission increased exponentially [27]. In this study, the index case (AH1) likely acquired the infection from the community as AH1 worked in a hospital ward with no known COVID-19 patients. The absence of epidemiological criteria contributed to a delay in diagnosing and isolating AH1 from the onset of symptoms. Individual compliance among HCWs to infection control measures is the first line of defense against a COVID-19 hospital outbreak during the mitigation phase [28]. Therefore, UMMC introduced entry screening visas for all HCWs, patients, and visitors to the hospital for self-screening for symptoms and risk factors. Additionally, a Special Staff Clinic was established to evaluate symptomatic HCWs who did not meet the COVID-19 screening criteria. An acute respiratory infection surveillance system was also implemented for both HCWs and patients.

Epidemiological case investigation and disease surveillance activities remain a cornerstone for outbreak management. Although molecular epidemiologic approaches are beneficial for elucidating transmission networks during outbreaks, the time and expense required for WGS are the limiting factors in most laboratories [29, 30]. WGS complemented the epidemiological investigation within our setting, as daily interactions between HCWs and patients on the ward and contact between HCWs were complex. There were multiple and repeated exposures that could not disentangle using conventional outbreak management strategies. WGS excluded four cases that were initially included as part of this cluster. A previous study of a COVID-19 outbreak on a cruise ship demonstrated the utility of WGS in establishing potential infection routes and linking transmission to a single source of infection [31]. Meanwhile, a prospective surveillance study that combined epidemiological and genomic analysis in examining hospital-associated COVID-19 cases had contributed to identifying transmission events within the healthcare setting. The evidence had informed the implementation of targeted approaches to prevent hospital-associated infections [7]. Thus, WGS plays a vital role in the surveillance of communicable diseases. Within the context of COVID-19, the increased availability of WGS and the public sharing of protocols and sequences have aided efforts to monitor transmission networks, circulating genotypes, and possible genetic determinants of virulence.

The findings from this study make several contributions to the current literature. First, the study highlights the contribution of integrating epidemiological investigation and WGS in understanding disease transmission in a hospital in an upper-middle income country. This study provides epidemiological evidence of a hospital-associated cluster involving HCWs, patients, and individuals in the community. The transmission of disease between the HCWs, patients, HCW-to-patient, and patient-to-HCW was speculated based on information gathered from the epidemiological investigation. However, the direction of transmissions within the cases remained unclear as there were multiple interactions between HCWs and patients during the outbreak. The evidence from WGS supported the epidemiological link between the cases with no known exposure to a COVID-19 case beyond the hospital setting or refuting some of the cases based on established contacts outside the cluster.

Second, it contributes to the growing body of literature on the transmission of COVID-19 from HCWs to the community, which remains limited despite accounting for a significant proportion of infections in this pandemic compared to earlier outbreaks of severe acute respiratory syndrome and the Middle East respiratory syndrome [32]. Numerous studies have focused on a single mode of transmission setting limited to either healthcare or non-healthcare settings among HCWs [32, 33]. Third, the study findings also emphasize the importance of limiting viral transmission within the hospital setting as onward community transmission of the disease was demonstrated from infected HCWs and patients to the community. Further propagation of disease could be prevented through good hospital governance in managing hospital-associated outbreaks. Specifically, it highlights the importance of a multidisciplinary approach, comprehensive infection control and prevention measures, and individual-level compliance towards ensuring a safe hospital population.

This study is not without limitations. First, the phylogenetic analysis was not conducted for all the cases due to resource constraints. The analysis nevertheless supported inclusion within this cluster of selected cases with clear transmission routes and excluded those of less certainty as they had contact with other cases separate from this cluster. Second, during the study period, SARs-CoV-2 had low genetic variability, and this may restrict the interpretation of phylogenetic clustering as non-directly related cases in the community may have similar viral genes. Third, we acknowledge the limitation of using diagnosis dates to determine the time of infection. It is especially true for individuals who had been readmitted to the hospital and tested positive for SARS-CoV-2. These patients may have had an undiagnosed asymptomatic infection during their last admission to the hospital. We also acknowledge the limitation of the inconclusive duration of the infectious period for COVID-19 and the possible underestimation of cases using 48 h before symptom onset. Besides, there is some uncertainty during the 48 h overlap when assessing directionality based solely on symptom onset for cases through forward and backward contact tracing due to the complexity of interactions between the HCWs and patients. The possible transmission of disease between the first to second generation cases was further clarified using detailed movement history and WGS.

## Conclusion

In conclusion, combining epidemiological case management with WGS helps delineate and control hospital outbreaks. Clear and consistent policies governing infection control, outbreak preparedness and response, and individual-level compliance are critical for preventing and minimizing the spread of COVID-19 in hospitals.

## Supplementary Information

**Additional file 1. **Risk assessment matrix.

**Additional file 2.** Demographic and epidemiological characteristics of patient-cases with community and hospital-onset of COVID-19.

**Additional file 3. **List of SARS-CoV-2 sequences generated in this study.

## Data Availability

The datasets used and/or analyzed during the current study are available from the corresponding author on reasonable request.

## Abbreviations

COVID-19: Coronavirus disease 2019
EMR: Electronic medical records
HCWs: Healthcare workers
IQR: Interquartile range
PCR: Polymerase chain reaction
PPE: Personal protective equipment
SARS-CoV-2: Severe acute respiratory syndrome coronavirus 2
UMMC: University Malaya Medical Centre
WGS: Whole genome sequencing
WHO: World Health Organization
