# Supplementary material for: Propagation of a hospital-associated cluster of COVID-19 in Malaysia
Source: BMC Infect Dis. 2021 Dec 9;21:1238. doi: 10.1186/s12879-021-06894-y (PMC8655495; doi:10.1186/s12879-021-06894-y)
Supplement: Supplementary file 1 — Additional file 1. Risk assessment matrix. [file 12879_2021_6894_MOESM1_ESM.pdf]

Additional File 1: Risk assessment matrix

**Category of risk exposure**	**Circumstances**
No identifiable risk	Healthcare worker without any direct close contact with a confirmed COVID-19 case<br>Did not enter active patient's area<br>Healthcare workers who adhered to the recommended personal protective equipment
Low risk	Healthcare workers who had close contact with patients with COVID-19<br>**AND**<br>Adhered to the recommended personal protective equipment
Medium risk	Healthcare workers who had prolonged close contact with patients with COVID-19<br>**AND**<br>Where the healthcare worker's mucous membranes or hands were exposed to potentially infectious materials for COVID-19
High risk	Healthcare workers who performed aerosol-generating procedures or were present in the room during the procedure or performed procedures of which respiratory secretions are likely to be poorly controlled on patients with COVID-19<br>**AND**<br>When the healthcare worker's eyes, nose, or mouth were not protected

Note: Procedures that could induce uncontrolled respiratory secretions include cardiopulmonary resuscitation, intubation and extubating, non-invasive ventilation, bronchoscopy, nebulizer therapy and sputum induction procedures.
Source: UMMC, 2020
