# Supplementary material for: Propagation of a hospital-associated cluster of COVID-19 in Malaysia
Source: BMC Infect Dis. 2021 Dec 9;21:1238. doi: 10.1186/s12879-021-06894-y (PMC8655495; doi:10.1186/s12879-021-06894-y)
Supplement: Supplementary file 2 — Additional file 2. Demographic and epidemiological characteristics of patient-cases with community and hospital-onset of COVID-19. [file 12879_2021_6894_MOESM2_ESM.pdf]

Additional File 2: Demographic and epidemiological characteristics of patient-cases with community and hospital-onset of COVID-19

	All patient-cases	Patient-cases with community-onset	Patient-cases with hospital-onset
Count, n	9	5	4
Age* in years, median [IQR]	80.0 [74.0, 81.0]	80.0 [77.0, 81.0]	77.5 [73.8, 8.0]
Female (%)	6 (66.7)	5 (100.0)	1 (25.0)
Symptomatic (%)	7 (77.8)	5 (100.0)	2 (50.0))
Fever	5 (55.6)	4 (80.0)	1 (25.0)
Cough	4 (44.4)	3 (60.0)	1 (25.0)
Sore throat*	1 (11.1)	1 (20.0)	-
Coryza	-	-	-
Anosmia	-	-	-
Dyspnoea	1 (1)	1 (20.0)	-
Gastrointestinal symptoms	3 (33.3)	3 (60.0)	-
Lethargy	-	-	-
Previous comorbidities* (%)	9 (100.0)	5 (100.0)	4 (100.0)
Diabetes mellitus	7 (77.8)	4 (80.0)	3 (75.0)
Hypertension	8 (88.9)	5 (100.0)	3 (75.0)
Cardiovascular disease	5 (55.6)	2 (40.0)	3 (75.0)
Hyperlipidaemia	6 (66.7)	3 (60.0)	3 (75.0)
Chronic kidney disease	3 (33.3)	2 (40.0)	1 (25.0)
History of cancer	-	-	-
Other comorbidities	7 (77.8)	3 (60.0)	4 (100.0)
Duration (in days), median [IQR]			
Symptom onset to diagnosis	1.0 [0.0, 2.0]	1.0 [0.0, 2.0]	1.0 [0.0, 3.0]
Symptom onset to admission to hospital	1.0 [0.0, 2.0]	1.0 [1.0, 2.0]	1.0 [0.0, 3.0]
Diagnosis to admission to hospital	0.0 [0.0, 0.0]	0.0 [0.0, 0.0]	0.0 [0.0, 0.0]
Admission to discharge (n=5)	33.0 [33.0, 39.0]	33.0 [33.0, 36.0]	23.0 [22.0, 24.0]

Notes:

Results are presented in median [interquartile range] for continuous variables and in the count (percentage) for categorical variables.

Gastrointestinal symptoms include nausea, vomiting, diarrhoea, and abdominal pain.
