# Supplementary material for: Propagation of a hospital-associated cluster of COVID-19 in Malaysia
Source: BMC Infect Dis. 2021 Dec 9;21:1238. doi: 10.1186/s12879-021-06894-y (PMC8655495; doi:10.1186/s12879-021-06894-y)
Supplement: Supplementary file 3 — Additional file 3. List of SARS-CoV-2 sequences generated in this study. [file 12879_2021_6894_MOESM3_ESM.pdf]

Additional File 3. List of SARS-CoV-2 sequences generated in this study.

Case code	Virus sequence name	GISAID accession ID	Collection date	Pangolin lineage	No. of raw reads	No. of mapped reads	Breadth of genome coverage (%)	Average depth of coverage
AH1	hCoV-19/Malaysia/0931/2020	EPI_ISL_501180	23-Mar-20	B.6.1	36522	36379	99.76	345.25
AH4	hCoV-19/Malaysia/2079/2020	EPI_ISL_501190	26-Mar-20	B.6.1	43363	43272	99.89	407.68
AH5	hCoV-19/Malaysia/2735/2020	EPI_ISL_501195	28-Mar-20	B.6.1	50720	50584	99.96	473.83
AH6	hCoV-19/Malaysia/2813/2020	EPI_ISL_501197	28-Mar-20	B.6.1	7908	7898	99.78	74.95
AH7	hCoV-19/Malaysia/6306/2020	EPI_ISL_501219	07-Apr-20	B.6.1	53512	53234	99.78	504.7
AH8	hCoV-19/Malaysia/3706/2020	EPI_ISL_501209	31-Mar-20	B.6.1	45002	44951	99.82	422.46
AH9	hCoV-19/Malaysia/3703/2020	EPI_ISL_501208	31-Mar-20	B.6.1	41330	41263	99.78	387.17
AH10	hCoV-19/Malaysia/2982/2020	EPI_ISL_506998	29-Mar-20	B.6.1	61653	56569	94.3	553.5
AP1	hCoV-19/Malaysia/2251/2020	EPI_ISL_501192	27-Mar-20	B.6.1	310702	308736	99.91	3012.73
AP2	hCoV-19/Malaysia/2065/2020	EPI_ISL_501189	26-Mar-20	B.6.1	39250	39151	99.78	366.14
AP3	hCoV-19/Malaysia/1713/2020	EPI_ISL_501186	25-Mar-20	B.6.1	42713	42636	99.82	400.8
AP4	hCoV-19/Malaysia/2363/2020	EPI_ISL_501194	27-Mar-20	B.6.1	47159	47049	99.78	443.53
AP7	hCoV-19/Malaysia/2101/2020	EPI_ISL_501191	26-Mar-20	B.6.1	164955	162989	99.91	1582.45
AP9	hCoV-19/Malaysia/5760/2020	EPI_ISL_506999	23-Apr-20	B.6.1	147641	82018	94.24	796.88
AG1	hCoV-19/Malaysia/5822/2020	EPI_ISL_501214	06-Apr-20	B.6.1	38876	38793	99.83	364.42
AF1	hCoV-19/Malaysia/5425/2020	EPI_ISL_501212	04-Apr-20	B.6.1	46610	46486	99.86	437.5
AN3	hCoV-19/Malaysia/3145/2020	EPI_ISL_501202	30-Mar-20	B.6.1	44186	44148	99.78	417.71
AX1	hCoV-19/Malaysia/3012/2020	EPI_ISL_501199	29-Mar-20	B.6	733738	724362	99.9	7119.37
AX2	hCoV-19/Malaysia/5906/2020	EPI_ISL_501215	06-Apr-20	B.6.6	40657	40579	99.89	380.13
AX3	hCoV-19/Malaysia/2063/2020	EPI_ISL_501188	26-Mar-20	B.6.6	40839	40663	99.76	382.8
AX4	hCoV-19/Malaysia/2811/2020	EPI_ISL_501196	28-Mar-20	B.6.6	45799	45679	99.89	426.84
